# Cervical Cancer Screening in HIV-Positive Women by Conventional Exfoliative Cytology and Liquid-Based Cytology

**DOI:** 10.7759/cureus.91118

**Published:** 2025-08-27

**Authors:** Himani Rai, Deepshikha Verma, Ravi Meena, Upasana Uniya, Neelesh Nagayach

**Affiliations:** 1 Department of Pathology, Mahamana Pandit Madan Mohan Malaviya Cancer Centre and Homi Bhabha Cancer Hospital, Varanasi, Varanasi, IND; 2 Department of Pathology, Atal Bihari Vajpayee Government Medical College, Vidisha, IND; 3 Department of Pathology, Indira Path Lab, Varanasi, IND; 4 Department of Pathology, Gandhi Medical College, Bhopal, IND; 5 Department of Pathology, Sunderlal Patwa Government Medical College, Mandsaur, Mandsaur, IND

**Keywords:** cervical cancer, cervical screening, conventional exfoliative cytology, hiv, liquid-based cytology, precancerous lesions

## Abstract

Background

Cervical cancer remains a significant public health challenge, particularly among women living with human immunodeficiency virus (HIV), due to their increased vulnerability to persistent human papillomavirus (HPV) infections. Early detection through effective screening methods such as conventional exfoliative cytology (CEC) and liquid-based cytology (LBC) can substantially reduce the burden of cervical cancer in this population.

Objective

In view of the combined burden of HIV infection and cervical cancer in India, the current study aims at screening for cervical cancer in HIV-positive women attending anti-retroviral therapy (ART) centers by conventional Pap smear and LBC and also compares the results of conventional exfoliative cytology to liquid-based cytology in Gandhi Medical College and Associated Hospitals, Bhopal.

Methods

This cross-sectional study was conducted on HIV-positive women attending a tertiary care center. Cervical samples were collected and analyzed using both CEC and LBC techniques. The results were evaluated based on cytological abnormalities, sample adequacy, and detection rates of precancerous lesions. Data were statistically analyzed to determine the comparative effectiveness of these two methods.

Results

Of the 250 HIV-positive women screened using conventional exfoliative cytology, epithelial abnormalities were detected in 25 (10.0%) cases. LBC demonstrated superior sample adequacy, with 230 out of 250 samples (92%) being satisfactory, compared to 200 out of 250 samples (80%) in CEC. Additionally, LBC showed higher sensitivity in detecting epithelial abnormalities, with 29 out of 29 cases (100.0%) correctly identified, compared to 25 out of 29 cases (86.2%) detected by CEC. Both methods demonstrated high specificity, with LBC identifying 221 out of 225 true negatives (98.2%) and CEC identifying 225 out of 225 true negatives (100.0%).

Conclusion

Liquid-based cytology is more effective than conventional exfoliative cytology in cervical cancer screening among HIV-positive women due to its higher sensitivity and better sample adequacy. Incorporating LBC into routine screening protocols for high-risk populations, such as HIV-positive women, could enhance early detection and improve clinical outcomes.

## Introduction

In developing countries, such as India, cervical cancer and human immunodeficiency virus (HIV) infection are important public health problems [1]. Cervical carcinoma is the fourth most common cancer in the world and the fourth leading cause of cancer-related fatalities in women, making it a significant public health issue. In 2018, an estimated 570,000 women were diagnosed with cervical cancer worldwide, and about 311,000 women died from the disease [2]. It is one of the major causes of cancer death among women aged 30-69 years, accounting for 17% of all cancer deaths. Cervical cancer is expected to affect one out of every 53 Indian women during their lifetime, compared to one out of every 100 women in more industrialized countries [3]. Almost 70% of the global burden of cervical cancer falls in areas with lower levels of development, and more than one-fifth of all new cases are diagnosed in India. For women in India, cervical cancer is the second most common cancer. Cervical cancer is also the second most common cause of death when both genders are combined [4].

The human papillomavirus (HPV) is the most common etiologic agent causing cervical cancer. HIV-positive women have a greater risk of cervical intraepithelial neoplasia (CIN) caused by HPV [1].

It is possible to identify intraepithelial lesions and cellular alterations in the cervix before a patient exhibits overt invasive cancer. Cervical screenings were thus implemented globally. Early in the 1940s, Dr. George N. Papanicolaou introduced the Pap stain and explained how vaginal smears could be made to screen for cervical malignancies. In both developed and developing countries, Pap smears have been used for decades to screen for cervical cancer and have been successful in reducing the prevalence of the disease by up to 75% [5]. The prevalence of cervical cancer-related death and morbidity has dramatically declined over time due to effective therapy, early identification by Pap smear screening and colposcopy, and preventive measures such as vaccinations [6].

Squamous cell carcinoma (SCC), high-grade squamous intraepithelial lesion (HSIL), and low-grade squamous intraepithelial lesions (LSIL) are among the abnormal Pap test outcomes that are more common in women with HIV [7]. Those with HIV had a 10-fold higher risk of abnormal Pap smears than those without HIV [8]. Therefore, upon initial diagnosis and when seeking prenatal care, the Centers for Disease Control and Prevention (CDC) advises all women infected with HIV to undergo a complete gynecological examination, which includes a Pap smear [9]. Cervical cancer is largely prevented if precursor lesions are found early and treated before they develop into cancer.

Although there is considerable debate about whether cervical cytology is an adequate screening tool for HIV-positive women, many studies have found that using conventional smearing procedures, both HIV-positive and HIV-negative women had equal sensitivities, specificities, and false-negative rates. Furthermore, cervical cytology remains the preferred screening method in HIV-positive women because colposcopy and tissue investigations have yet to be proven to be as cost-efficient as screening techniques [10].

Due to poor quality samples and processing and the presence of obscuring blood, inflammation, and overlapping epithelial cells, the sensitivity of a standard Pap smear is lowered to less than 50%. As a result, false-positive and false-negative test findings were produced. In the 1990s, enhanced, newer-technology, liquid-based cytology was launched in the United States to address these issues.

A thin, homogeneous layer of cells is generated on a slide using liquid-based cytology (LBC), which entails washing the sample instrument in a liquid medium to produce a cell suspension. By removing obscuring substances such as mucus, blood, and debris, this method improves slide clarity and cuts down on interpretation time. LBC has a number of benefits over traditional Pap smears, including a decreased incidence of insufficient samples and the preservation of enough cellular material for further testing, such as the molecular detection of infectious agents such as *Neisseria gonorrhoeae*, *Chlamydia trachomatis*, and human papillomavirus (HPV).

Liquid-based Pap tests have been shown to have lower frequencies of atypical squamous cells of uncertain significance (ASCUS) and higher identification of squamous intraepithelial lesions as compared to conventional smears in conventional exfoliative cytology (CEC). Many studies have compared LBC to conventional Pap, with the majority showing LBC reporting better sensitivity for detecting pathological alterations and a higher proportion of slides suitable for assessment [11].

In view of the combined burden of HIV infection and cervical cancer in India, the present study aims at cervical cancer screening in HIV-infected women attending anti-retroviral therapy (ART) centers by conventional Pap smear and liquid-based cytology and also compares the results of conventional exfoliative cytology to liquid-based cytology in Gandhi Medical College and Associated Hospitals, Bhopal.

## Materials and methods

A prospective observational study was conducted over a period of 18 months (from 1 January 2020 to 1 June 2021) to screen for cervical cancer in 250 HIV-positive women attending the ART center of Gandhi Medical College and Hamidia Hospital, Bhopal. Screening was performed using both conventional exfoliative cytology and liquid-based cytology (LBC).

The study received approval from the Institutional Ethics Committee of Gandhi Medical College (approval number: 631/MC/IEC/2020), and written informed consent was obtained from all participants. Confidentiality was maintained throughout the study. Inclusion criteria consisted of all HIV-positive women aged 18-65 years attending the ART center during the study period. Exclusion criteria included HIV-negative women, those who declined cervical examination and screening, pregnant women, women with a prior hysterectomy, those with active genital bleeding, and patients with known cervical malignancy or already under treatment for cervical intraepithelial neoplasia.

A detailed medical and gynecological history was recorded for each participant. All underwent a general physical examination, pelvic examination, and per speculum inspection of the cervix. Patients were positioned in the lithotomy position, and cervical samples were collected using Ayre’s spatula and an endocervical brush. For the conventional smear, cells from both devices were immediately transferred to pre-labeled slides and fixed in 95% ethanol. For liquid-based cytology, the cervical brush head was detached and placed in an EziPREP (Clintech Pvt. Ltd., Pune, India) LBC vial containing 20 mL of alcohol-based fixative solution.

Sample size calculation

The sample size was determined based on the prevalence of cervical abnormalities among HIV-positive women from previously published studies. Assuming a confidence level of 95%, the sample size was calculated using the formula n=Z^2.^p⋅(1−p)/d^2^, where n=required sample size, Z-value corresponding to the desired confidence level (1.96 for 95%); p=estimated prevalence of cervical abnormalities; and d=absolute precision (margin of error).

Based on this calculation, a sample size of N was required. To account for nonresponse or inadequate samples, the sample size was increased to 250 participants. A sample size of 250 was chosen based on feasibility, the availability of patients at the ART center, and comparable sample sizes in prior literature. The sample was considered adequate for detecting significant differences between the two cytological methods with sufficient power.

Statistical analysis

Data were entered into Microsoft Excel (Microsoft Corp., Redmond, WA) and analyzed using SPSS software version 25.0 (IBM Corp., Armonk, NY). The following statistical analyses were performed: descriptive statistics (frequency, percentages, and mean±SD) were used for demographic and clinical data. The chi-square test was used to compare categorical variables between conventional and LBC smears (e.g., epithelial abnormalities and sample adequacy). Cross-tabulation was done to assess concordance between CEC and LBC findings.

Diagnostic performance metrics such as sensitivity, specificity, positive predictive value (PPV), negative predictive value (NPV), and the accuracy of LBC were calculated by considering CEC as the reference standard. A p-value of <0.05 was considered statistically significant.

## Results

A total of 250 HIV-positive women attending the ART center of Gandhi Medical College and Hamidia Hospital were screened for cervical cancer during a period of 18 months. The cervical samples were processed by conventional methods and liquid-based cytology. Both cervical smears were reported using the Bethesda System of Cervical Cytology 2014, and results were compared. To minimize bias, the cytopathologists were blinded to the method of preparation (conventional smear versus liquid-based cytology), as well as to the patients’ clinical details.

Age-wise distribution was shown in Table 1.

**Table 1:** Age-wise distribution of cases

Age Group	Frequency	Percent
20-29 Years	42	16.8
30-39 Years	111	44.4
40-49 Years	66	26.4
50-59 Years	22	8.8
≥60 Years	9	3.6
Total	250	100.0

The majority of participants were in the 30-39 years age group, followed by those in the 40-49 years age group. A small proportion of women (n=9) were aged 60 years or above.

On per speculum examination, the majority of women (n=154, 61.6%) had a healthy cervix and vagina with no discharge observed. A total of 84 women (33.6%) had a healthy cervix and vagina, but vaginal discharge was present. Cervical erosion was noted in 12 women (4.8%). Results of conventional Pap according to the Bethesda System of Cervical Cytology 2014 were shown in Table 2.

**Table 2:** Results of conventional Pap according to the Bethesda System of Cervical Cytology 2014 NILM, negative for intraepithelial malignancy; ASCUS, atypical squamous cells of undetermined significance; HSIL, high-grade squamous intraepithelial lesion; ASC-H, atypical squamous cells, cannot exclude HSIL; LSIL, low-grade squamous intraepithelial lesion; SCC, squamous cell carcinoma

Conventional Smear	Frequency	Percent
Inadequate	7	2.8
NILM (No Inflammation and No Epithelial Abnormalities)	95	38.0
NILM, Inflammatory	83	33.2
NILM, Bacterial Vaginosis	27	10.8
NILM, Bacterial Vaginosis, Trichomonas	1	0.4
NILM, Trichomonas	4	1.6
NILM, *Candida*	3	1.2
NILM, *Actinomyces*	1	0.4
NILM, Atrophic	4	1.6
ASCUS	6	2.4
ASC-H	1	0.4
LSIL	2	0.8
HSIL	13	5.2
SCC	3	1.2
Total	250	100.0

In conventional cytology, 2.8% of smears (seven out of 250) were unsatisfactory for evaluation. Thirty-eight percent of cases (95 out of 250) had no inflammation and no epithelial abnormalities. Of cases, 33.2% (83 out of 250) were categorized under the negative for intraepithelial malignancy (NILM) inflammatory category. Under the NILM microorganism category, 10.8% of cases (27 out of 250) had bacterial vaginosis, 1.6% (four cases) had trichomonas infection, and only one case (0.4%) had a mixed infection of bacterial vaginosis and trichomonas. Of cases, 1.2% (three cases) had *Candida*, and one case had bacteria morphologically consistent with *Actinomyces*. Among epithelial abnormalities, 2.4% of cases (six out of 250) were categorized under the atypical squamous cells of undetermined significance (ASCUS) category; 0.4% (one case) as atypical squamous cells, cannot exclude HSIL (ASC-H); 0.8% (two cases) as low-grade squamous intraepithelial lesion (LSIL), 5.2% (13 cases out of 250) as high-grade squamous intraepithelial lesion (HSIL), and 1.2% (three cases) as squamous cell carcinoma.

Findings of LBC and conventional cytology were compared in Table 3 and Table 4.

**Table 3:** Distribution of LBC diagnoses across CEC categories NILM, negative for intraepithelial malignancy; ASCUS, atypical squamous cells of undetermined significance; HSIL, high-grade squamous intraepithelial lesion; ASC-H, atypical squamous cells, cannot exclude HSIL; LSIL, low-grade squamous intraepithelial lesion; SCC, squamous cell carcinoma; CEC, conventional exfoliative cytology; LBC, liquid-based cytology

Conventional Cytology	Inadequate (n, %)	NILM (n, %)	ASCUS (n, %)	ASC-H (n, %)
Inadequate	1 (0.4%)	5 (2.0%)	0 (0.0%)	0 (0.0%)
NILM	0 (0.0%)	215 (86.0%)	1 (0.4%)	0 (0.0%)
ASCUS	0 (0.0%)	0 (0.0%)	1 (0.4%)	0 (0.0%)
ASC-H	0 (0.0%)	0 (0.0%)	0 (0.0%)	1 (0.4%)
LSIL	0 (0.0%)	0 (0.0%)	0 (0.0%)	0 (0.0%)
HSIL	0 (0.0%)	0 (0.0%)	0 (0.0%)	0 (0.0%)
SCC	0 (0.0%)	0 (0.0%)	0 (0.0%)	0 (0.0%)
Total	1 (0.4%)	220 (88.0%)	2 (0.8%)	1 (0.4%)

**Table 4:** Distribution of LBC diagnoses across CEC categories Pearson chi-square test: χ²=773.84, df=36, and p<0.001. This indicates a statistically significant association between conventional cytology and LBC findings NILM, negative for intraepithelial malignancy; ASCUS, atypical squamous cells of undetermined significance; HSIL, high-grade squamous intraepithelial lesion; ASC-H, atypical squamous cells, cannot exclude HSIL; LSIL, low-grade squamous intraepithelial lesion; SCC, squamous cell carcinoma; LBC, liquid-based cytology; CEC, conventional exfoliative cytology; df, degrees of freedom

Conventional Cytology	LSIL (n, %)	HSIL (n, %)	SCC (n, %)	Total (n, %)
Inadequate	0 (0.0%)	1 (0.4%)	0 (0.0%)	7 (2.8%)
NILM	2 (0.8%)	0 (0.0%)	0 (0.0%)	218 (87.2%)
ASCUS	3 (1.2%)	2 (0.8%)	0 (0.0%)	6 (2.4%)
ASC-H	0 (0.0%)	0 (0.0%)	0 (0.0%)	1 (0.4%)
LSIL	2 (0.8%)	0 (0.0%)	0 (0.0%)	2 (0.8%)
HSIL	0 (0.0%)	11 (4.4%)	2 (0.8%)	13 (5.2%)
SCC	0 (0.0%)	0 (0.0%)	3 (1.2%)	3 (1.2%)
Total	7 (2.8%)	14 (5.6%)	5 (2.0%)	250 (100.0%)

The sensitivity and specificity of LBC, when compared to conventional smear, are shown in Table 5.

**Table 5:** Sensitivity and specificity of liquid-based cytology compared to conventional cytology A Pearson chi-square test was performed to assess the association between the detection of epithelial abnormalities by liquid-based cytology (LBC) and conventional cytology. The test revealed a statistically significant association (χ²=211.686, df=1, and p<0.001), indicating a strong correlation between the two methods in identifying abnormal cytological findings df: degrees of freedom

Parameter	Value
Sensitivity	100.0%
Specificity	98.2%
Positive Predictive Value (PPV)	86.2%
Negative Predictive Value (NPV)	100.0%
Accuracy	98.4%

For the diagnosis of cervical epithelial abnormalities, when compared to conventional Pap, LBC had a sensitivity of 100%, a specificity of 98.2%, a positive predictive value of 86.2%, a negative predictive value of 100%, and an accuracy of 98.4%.

**Figure 1:**
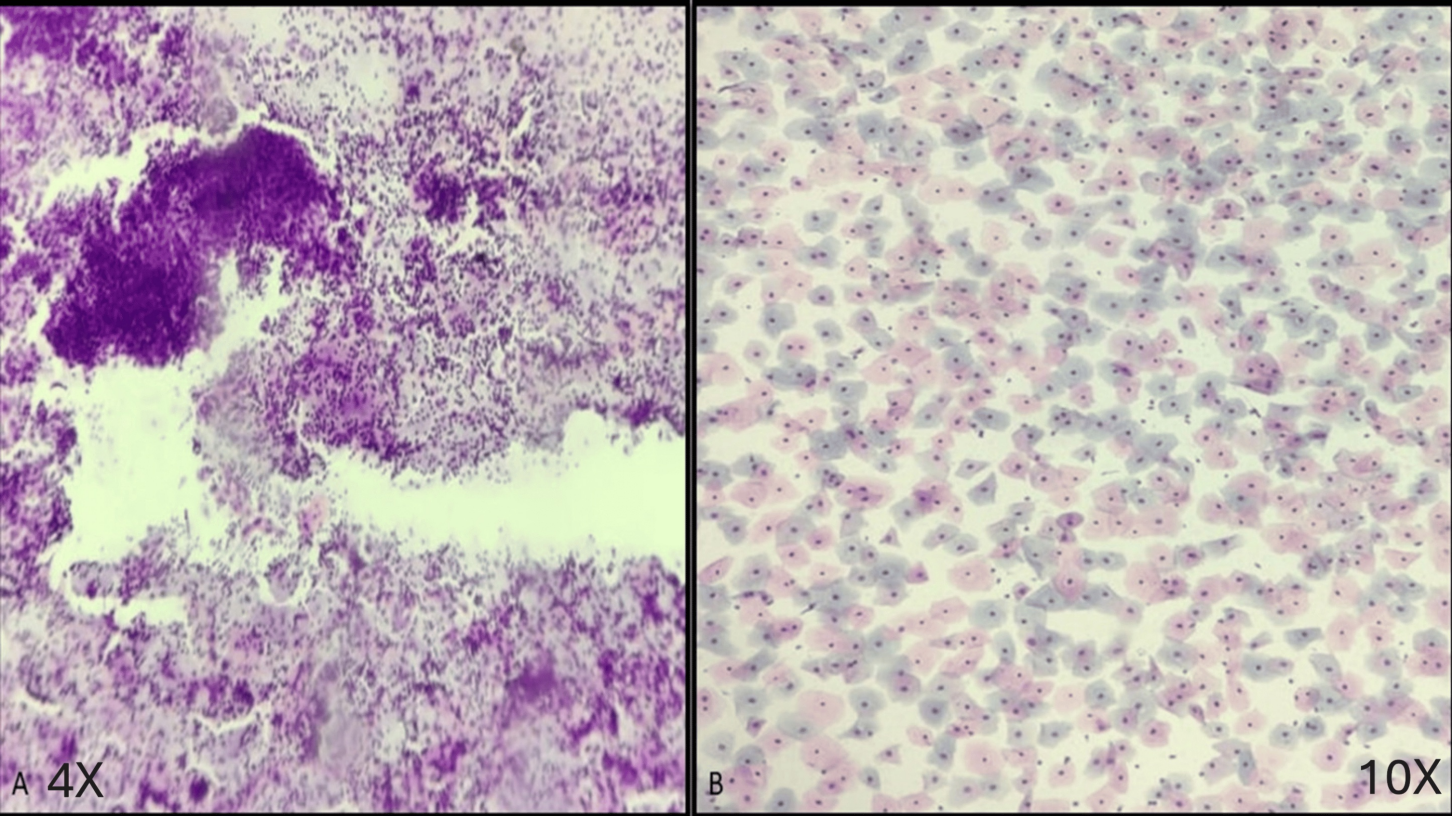
Scanner view of conventional smear (A) and liquid-based cytology (LBC) smear (B) (A) Conventional smear (scanner view, Pap stain) showing the overlapping of squamous cells and the presence of obscuring factors such as inflammation and hemorrhage. (B) LBC smear (low-power view, Pap stain) showing a monolayer of cells with a clear background; cytomorphological details can be clearly interpreted

A comparison of conventional smear to liquid-based cytology is shown in Figure 1 (Figure 1A shows a scanner view of a conventional smear, while Figure 1B shows a low-power view of an LBC smear).

## Discussion

With the increasing prevalence of HIV and its well-established link to cervical cancer and its precursor lesions, this prospective study is important in evaluating and comparing the efficacy of conventional exfoliative cytology and liquid-based cytology (LBC) in screening HIV-positive women for cervical abnormalities.

A total of 250 HIV-positive women attending the ART center of our hospital were screened using both cytological methods. The findings from conventional cytology revealed epithelial abnormalities in 10% of cases, including ASCUS (2.4%), ASC-H (0.4%), HSIL (5.2%), LSIL (0.8%), and squamous cell carcinoma (1.2%). In comparison, liquid-based cytology detected epithelial abnormalities in 11.6% of participants, comprising ASCUS (0.8%), ASC-H (0.4%), LSIL (2.8%), HSIL (5.6%), and squamous cell carcinoma (2%).

These results are comparable to those reported by Chalermchockcharoenkit et al. (2011), who screened 821 HIV-positive women and observed a 15.4% prevalence of cervical squamous cell abnormalities: ASCUS (2.8%), ASC-H (0.6%), LSIL (8.5%), and HSIL (3.5%) [12]. Similarly, Prabha Devi and Bindhu Priya (2013) found a 7.17% prevalence of abnormal Pap smears in HIV-positive women, which is approximately twice the risk compared to the general population [13]. In an earlier study, Maiman (1998) reported that 30%-60% of Pap smears from HIV-positive women showed cytological abnormalities and 15%-40% demonstrated evidence of dysplasia [14].

The variation in prevalence rates across studies may be attributed to differences in sample sizes, sociodemographic backgrounds, clinical stages of HIV infection, and age distribution of the study populations. These factors must be considered when comparing outcomes and generalizing findings across populations.

Comparison of liquid-based cytology to conventional exfoliative cytology

Comparison to Liquid-Based Cytology (LBC)

In a previously published study using the same cohort, liquid-based cytology (LBC) was used to evaluate cervical samples from the same group of 250 HIV-positive women [15]. Compared to conventional exfoliative cytology, LBC showed a higher rate of detection for epithelial abnormalities (11.6% in LBC versus 10% in CEC), greater sample adequacy (99.6% in LBC versus 97.2% in CEC), and improved clarity of cytomorphological details due to monolayer presentation and the absence of obscuring factors such as hemorrhage and inflammation.

Specifically, LBC identified LSIL in 2.8%, HSIL in 5.6%, and SCC in 2% of cases compared to 0.8%, 5.2%, and 1.2% in CEC, respectively. Moreover, fewer smears were deemed inadequate in LBC (0.4%) compared to CEC (2.8%). These findings highlight the superiority of LBC in both diagnostic accuracy and the ease of interpretation, although cost and accessibility remain important limiting factors in many resource-limited settings.

The cross-tabulation and statistical comparison (Pearson chi-square=773.84, p<0.001) between CEC and LBC showed significant differences in diagnostic yield. When conventional cytology was taken as the reference, LBC demonstrated a sensitivity of 100%, a specificity of 98.2%, and a diagnostic accuracy of 98.4%.

Comparison of screening time

In the present study, the average time taken for screening and reporting a CP smear was six minutes compared to two minutes for an LBC smear. This was because of the monolayer of the cells in LBC and fewer obscuring factors such as hemorrhage, inflammation, and mucus in LBC when compared to conventional smears. So, LBC smears were easier to screen and report when compared to conventional smears.

The findings were similar to Gupta et al. [16] and Cheung et al. [17], who also observed less screening time for LBC smears (2-1 minutes) when compared to conventional smears (8-4 minutes).

Comparison of smear

In the present study, a low-cost LBC EziPREP was used, which offered double smears in a single slide with a 16 mm diameter each. So, we got a total of 32 mm of screening area in this LBC. This is more when compared to other first-generation USFDA-approved LBCs, “SurePath” and “ThinPrep,” which provided single smears with 13 mm and 20 mm screening areas, respectively.

In LBC smears, cellular overlapping and crowding were less, whereas this was a common occurrence in conventional Pap smears. LBC smears had a clean background, with polymorphs aggregating to form easily discernible clusters, and mucus was substantially reduced in these smears. On the other hand, conventional smears had polymorphs all across the smear. The amount of RBCs was higher, and this interfered with smear interpretation in conventional smears.

Similar findings were observed in studies by Gupta et al. [16], Sulochana et al. [18], and Nandini et al. [19], who also found a clean background in LBC as compared to conventional smear.

Comparison of adequacy

In the present study, the percentage of satisfactory smears in conventional Pap was 97.2%, as compared to that of 99.6% satisfactory smears in LBC preparation. Seven cases were reported as inadequate in conventional smear, and one case was reported as inadequate in Pap smear. Out of seven inadequate cases in conventional smear, three cases were reported as NILM in LBC, two cases as bacterial vaginosis, and one case as HSIL in LBC.

In all these studies, LBC had a higher number of satisfactory smear rates when compared to conventional Pap. In the present study, the reason for the greater number of unsatisfactory smears in conventional Pap was due to thick and hemorrhagic smears and reduced cellularity when compared to LBC preparation, which was similar to the study by Monsonego et al. [20]. In addition, only 20% of the cells collected on the brush are smeared on the slide in conventional Pap, resulting in a lower number of cells being transferred to the smear for screening [21].

According to Sherwani et al., in LBC, cytolysis and drying artifacts are minimal or absent due to quick fixation in a liquid fixative and less limited components such as inflammatory cells, blood, and mucus, but in conventional Pap, cytolysis and drying artifacts are due to thick smears [22].

In the present study, 2.5% (six out of 250) of cases were diagnosed as ASCUS in conventional smears, whereas 0.8% (two out of 250) of cases were diagnosed as ASCUS in LBC. One case diagnosed as NILM in a conventional smear was diagnosed as ASCUS in LBC. Out of six cases diagnosed as ASCUS in conventional smear, three cases were upgraded to LSIL in LBC, and two cases were upgraded to HSIL in LBC. This was because of the clearer background, less cellular overlapping, and better interpretation of cytological and nuclear details in LBC as compared to conventional smears. This indicates that LBC may detect higher-grade lesions that are underdiagnosed on conventional smears. From a clinical perspective, such upgrades are significant, as they may prompt earlier colposcopic evaluation and closer follow-up, ensuring timely intervention and potentially improving patient outcomes.

Other similar studies by Shobana and Saranya [23], Zhu et al. [5], and Qureshi et al. [24] also found a greater number of ASCUS in conventional smears as compared to LBC.

In contrast, Hatch [25], Guidos and Selvaggi [26], İlter et al. [27], and Bolick and Hellman [28] had a greater number of ASCUS diagnoses in LBC when compared to conventional smears. The present study shows a sensitivity of 86.2% and a specificity of 100% in detecting epithelial abnormalities for conventional Pap smears and a sensitivity of 100% and a specificity of 98.2% in detecting epithelial abnormalities for liquid-based cytology. Previous studies found that LBC had a greater sensitivity and detection rate than conventional smears.

The significant reduction in the number of insufficient samples seen in the present study, from 2.8% in conventional smears to 0.4% in LBC, and the easy pickup of organisms and epithelial abnormalities in LBC compared to conventional cytology suggest that LBC should be used as the preferred screening technology in HIV-positive women, at least where the additional expense can be justified.

Limitations

As a tertiary care center situated in the capital of the state, it covers the usual population surrounding it, which is usually urban, suburban, and near peripheral areas, but there is a huge periphery that still remains unscreened under this study. Future studies could focus on community-based or outreach screening programs to include these underserved populations, thereby improving the coverage and early detection of cervical neoplasia in high-risk groups such as HIV-positive women.

This study had a relatively smaller sample size, which may limit the precision of our findings and affect the generalizability to the broader HIV-positive population. Consequently, the exact prevalence of cervical epithelial abnormalities could not be determined. Additionally, the absence of concurrent HPV testing further restricts the ability to correlate cytological findings with viral infection status. Future studies with larger cohorts and integrated HPV testing are recommended to more accurately establish prevalence and improve risk stratification in this high-risk population.

## Conclusions

The prevalence of cervical epithelial abnormalities is high in HIV-positive women; therefore, all HIV-positive women should be regularly screened for cervical cancer. To provide effective screening in this population, low-cost liquid-based cytology should be used to avoid repeated calling of the patients and to get better results.
